# Interleukin-10 Promotes Porcine Circovirus Type 2 Persistent Infection in Mice and Aggravates the Tissue Lesions by Suppression of T Cell Infiltration

**DOI:** 10.3389/fmicb.2019.02050

**Published:** 2019-09-10

**Authors:** Qian Du, Huan Zhang, Mingrui He, Xuan Zhao, Jia He, Beibei Cui, Xuefeng Yang, Dewen Tong, Yong Huang

**Affiliations:** ^1^College of Veterinary Medicine, Northwest A&F University, Yangling, China; ^2^College of Life Science, Northwest A&F University, Yangling, China

**Keywords:** porcine circovirus type 2, interleukin-10, lymphocytes, T cells, chemokines

## Abstract

Interleukin (IL)-10, as a key anti-inflammatory cytokine, increases during porcine circovirus type 2 (PCV2) infection, but the role of IL-10 in the process remains to be defined. In the present study, using an IL-10 deficient mice model, we found that IL-10 deficiency prevented the reduction of splenic lymphocytes (CD45^+^ cells) induced by PCV2 and promoted CD4^+^ and CD8^+^ T cell infiltration in lungs through inducting more T cell chemokines (CCL3, CXCL9, and CXCL10). Simultaneously, PCV2 infection induced a significant increase of pro-inflammatory cytokines and PCV2-specific antibodies in IL-10 deficient mice than in wild-type mice, resulting in a lower viral load in lung and a milder lung lesion in IL-10 deficient mice relative to wild-type mice. Moreover, the amounts of pulmonary CD4^+^ and CD8^+^ T cells were all inversely correlated with the lung lesions, as well as the viral load of PCV2. These results demonstrate that PCV2 infection employs IL-10 to block the transfer of T cells to the lungs of mice, and IL-10 attenuates the production of pro-inflammatory cytokines and PCV2-specific antibodies. The lack of T cell infiltration, pro-inflammatory cytokines, and PCV2-specific antibodies promote PCV2 replication, leading to a more severe lung lesion in mice.

## Introduction

Porcine circovirus type 2 (PCV2) is the primary causative agent of porcine circovirus disease (PCVD) in piglets (Segalés, 2015). The PCVD symptoms are characterized by weight loss, interstitial pneumonia, lymphocytes depletion, and multisystemic wasting in pigs (Segalés, 2012). PCV2 is considered to mainly affect the immune system and cause immunosuppression, which lead the piglets to become susceptible to other pathogens, such as porcine reproductive respiratory syndrome virus (PRRSV), porcine parvovirus (PPV), *Haemophilus parasuis*, or *Mycoplasma hyopneumoniae* (Afghah et al., 2017; Niederwerder, 2017; Du et al., 2018). The coinfection with other pathogens results in severe clinical diseases and leads to serious economic losses in world pig industry (Denner and Mankertz, 2017).

IL-10 plays an important role in protecting the host from inflammation injury by regulating the balance of immune response (Couper et al., 2008). IL-10 mainly limits the activation and proliferation of both the innate and the adaptive immune cells to maintain homeostasis (O’Farrell et al., 1998; Couper et al., 2008; Pino-Martinez et al., 2019). The role of IL-10 is vitally important in protecting the host from inflammation-associated immunopathology, autoimmunity, and allergy by ameliorating the excessive CD4^+^ and CD8^+^ T cell responses (Couper et al., 2008). However, IL-10 could be employed to escape host defense by some viruses. Previous studies demonstrated that PCV2 infection induces IL-10 overexpression in pigs, and we previously proved that PCV2 infection induces a high level of IL-10 production in porcine alveolar macrophages (Kekarainen et al., 2008; Du et al., 2016). Although the PCV2-induced IL-10 production is considered to be associate with the thymic depletion of pigs (Doster et al., 2010), the roles of IL-10 in the process of PCV2 infection remain to be defined.

Mouse has been widely used as an infection model to study virus-host interactions. PCV2 is previously reported to replicate in BALB/c mice, and the virus can be detected in lymphoid tissues, livers, spleens, and thymus (Kiupel et al., 2001). PCV2 is also confirmed to replicate and transmit in CRL: NMRI BR mice, Kunming mice, and CH3/Rockefeller mice (Csagola et al., 2008; Deng et al., 2013; de Castro et al., 2015). Besides, the immunomodulatory effect of PCV2 on DCs was investigated in BALB/c mice model (Wang et al., 2017). In this study, the wild-type C57BL/6 mice and *il10* knockout (*il10*^−/−^) C57BL/6 mice infected with PCV2 were used to investigate the role of IL-10 in the depletion of lymphocytes, especially T cells during PCV2 infection. These results would give a further understanding of the pathogenic mechanism of PCV2.

## Materials and Methods

### Ethics Statement

All animal experiments were approved by the Institutional Animal Care and Use Committee (IACUC) of Forth Military Medical University, China (permit number: 18017) and were performed according to the Animal Ethics Procedures and Guidelines of the People’s Republic of China. No other specific permissions were required for these activities. This study did not involve endangered or protected species.

### Virus, Reagents, and Antibodies

PCV2 (strain: PCV2b, GenBank: MH492006) was stocked in our laboratory and propagated in PK15 cells. The copy number of PCV2 was measured by quantitative polymerase chain reaction (PCR) as previously described (Wang et al., 2018). The flow cytometry buffer, APC anti-mouse CD45 (103112, 30-F11), FITC anti-mouse CD3 (100204, 17A2), PE anti-mouse CD4 (100408, GK1.5), and PE anti-mouse CD8a (100707, 53–6.7) monoclonal antibodies were purchased from BioLegend. The rabbit anti-PCV2 Rep serum was obtained in our lab (Chen et al., 2015).

### Animal, Housing, and Experiment Design

Six-week-old healthy C57BL/6 female mice were purchased from Lab Animal Centre of Forth Military Medical University and *il10*^−/−^ female mice (B6.129P2-Il10^tm1^/Nju) originally from the Jackson Laboratory, USA (Stock No: J002251) were purchased from Model Animal Research Center of Nanjing University, China. We chose only female mice for sex uniformity in this study. The *il10*^−/−^ mice are of C57BL/6 background and homozygous for the *Il10*^tm1Cgn^ targeted mutation. All the mice were housed under same conditions and treated in a similar way. In each test, 20 wild-type mice were divided into four groups and 20 *il10*^−/−^ mice were also divided into four groups, and each group contained five mice. Three wild-type mice groups were infected with 8.95 × 10^7^ copies/kg body weight PCV2 oronasally in 100 μl of DMEM, whereas one wild-type mice group was mock infected with the same volume of DMEM alone. Also, one *il10*^−/−^ mice group was mock infected, and other three groups were infected with the same dose of PCV2. The three PCV2-infected groups were euthanized for further analysis at 7, 14, and 28 days post-infection (d.p.i.), respectively. The mock-infected mice were euthanized at 28 d.p.i. The blood from the mice was used to analyze IL-10 production and antibodies. The whole spleens were collected for lymphocytes measurement. The lungs were separated for different detections, the right lungs were used for lymphocytes and antibodies measurements, and left lungs were further cut into two parts. One part of the left lungs was used for histopathological examination, and the other part was used for chemokine mRNA, viral load, and protein expression analyses. The animal experiments were repeated three times.

For cytokine measurements, 40 wild-type mice were divided into two groups, one group was mock infected and the other was PCV2 infected. Also, 40 *il10*^−/−^ mice were also divided into two groups, one group was mock infected and the other was PCV2 infected. Mock-infected and PCV2-infected mice were euthanized at 0, 1, 3, and 6 h post infection (*n* = 5, for each time and each group). Serum samples were collected to measure the production of cytokines.

For the mixed feeding experiment, six wild-type mice and six *il10*^−/−^ mice were fed in one container, the mice were randomly infected with different doses of PCV2. At 28 d.p.i., the mice were euthanized for pathological changes, flow cytometry, and qPCR analysis.

### Enzyme-Linked Immunosorbent Assay

The cytokines IL-10 (431414, BioLegend), IL-2 (431004, BioLegend), IL-6 (431304, BioLegend), IL-12 (433604, BioLegend), TNF-α (430904, BioLegend), IFN-α (BMS6027, Invitrogen), and IFN-γ (430804, BioLegend) were measured by the commercial enzyme-linked immunosorbent assay (ELISA) kits following the manufacturer’s instruction. The IgG contents were detected by an automatic biochemical analyzer (BS-180, mindray, China). The PCV2-specific antibody production was detected as the previously reported (Li et al., 2016), that the purifed recombinant Cap protein without nuclear localization signal expressed by pET-32a were used to coat 96-well plates, the coating plates were used for the detection. For pulmonary antibodies measurement, the right lungs were cut into pieces in tubes, and the supernatants after centrifugation were used for analyses.

### Preparation of Lymphocytes

For the pulmonary lymphocytes, the right lungs were resuspended with PBS containing 0.1% Collagenase type I (SCR103, Sigma-Aldrich), 0.05% DNase I (D5025, Sigma-Aldrich), and 5% FBS (13011-8611, Tianhang Biotechnology). After incubation at 37°C for 45 min, the tissues were pestled and filtrated with a 200-mesh sieve. The cells were centrifugated and resuspended with PBS, and then treated by Lymphocyte Separation Medium (P8620, Solarbio) as per the manufacturer’s instruction. For the splenic lymphocytes, the spleens were pushed through a 200-mesh sieve to obtain single-cell suspensions, and the suspensions were also treated by Lymphocyte Separation Medium.

### Flow Cytometry

The lymphocytes were blocked with FcR Blocking Reagent (130-092-575, Miltenyi Biotec) in dark for 20 min, and the cells were further incubated with conjugated antibodies in dark for 30 min. The APC-conjugated anti-CD45 antibody was used to stain total lymphocytes. The FITC anti-CD3 antibody and PE anti-CD4 antibody were used to stain CD4^+^ T cells among the CD45^+^ cells, while the FITC anti-CD3 antibody and PE anti-CD8 antibody were used to stain CD8^+^ T cells among the CD45^+^ cells. After the wash process, the stained cells were analyzed using BD Accuri C6 Flow Cytometer. The gate strategies are shown in Supplementary Figure S1.

### Quantitative Polymerase Chain Reaction

A portion of the lungs of mice was frozen at −80°C until use for quantitative PCR. Total RNA was extracted by TRIzol (15596026, Invitrogen) following the manufacturer’s instruction and the purity and concentration of the RNA were checked by NanoDrop 2000 Spectrophotometer (Thermo, USA). The reverse transcriptions of 2 μg of RNA were performed with random primers by M-MLV (28025013, Invitrogen) as per the manufacturer’s instruction. Quantitative PCR analyses were performed on the IQ5 real-time PCR System (Bio-Rad) using SYBR Premix Ex Taq II (RR820A, TAKARA) and calculated by 2^−ddCt^. 20 ng cDNAs from the lungs were used in the quantitative PCR and the reaction was perform as 95°C 30 s; 95°C 5 s, 60°C 30 s, 40 cycles; 65–95°C, increment 0.5°C. The primers are listed in Table 1.

**Table 1:** Primers for quantitative PCR.

Gene (GenBank no.)	Primers	Sequences	References
CCL3 (NM_011337)	Forward	TTCTCTGTACCATGACACTCTGC	Zhang et al., 2018
	Reverse	CGTGGAATCTTCCGGCTGTAG	
CCL4 (NM_013652)	Forward	TTCCTGCTGTTTCTCTTACACCT	Zhang et al., 2018
	Reverse	CTGTCTGCCTCTTTTGGTCAG	
CCL5 (NM_013653)	Forward	GCTGCTTTGCCTACCTCTCC	Zhang et al., 2018
	Reverse	TCGAGTGACAAACACGACTGC	
CCL8 (NM_021443)	Forward	GCTGTGGTTTTCCAGACCAA	Lopes et al., 2018
	Reverse	GAAGGTTCAAGGCTGCAGAA	
CXCL9 (NM_008599)	Forward	TGCACGATGCTCCTGCA	Amano et al., 2018
	Reverse	AGGTCTTTGAGGGATTTGTAGTGG	
CXCL10 (NM_021274)	Forward	GCCGTCATTTTCTGCCTCATC	Strehlitz et al., 2018
	Reverse	TAGGCTCGCAGGGATGATTTC	
CXCL11 (NM_019494)	Forward	CGAGTAACGGCTGCGACAAA	Strehlitz et al., 2018
	Reverse	TCACAGTCAGACGTTCCCAG	
CX3CL1 (NM_009142)	Forward	ACGAAATGCGAAATCATGTGC	Okuma et al., 2017
	Reverse	CTGTGTCGTCTCCAGGACAA	
β-actin (NM_007393)	Forward	GCGCGGCTACAGCTTCACCA	Luo et al., 2017
	Reverse	GGGCAGCGGAACCGCTCATT	

### Histopathological Examination

The separated lungs were immediately fixed by formalin and immersed in paraffin. Then, 5-μm-thick sections of the lungs were stained with hematoxylin and eosin (H&E) for examination. The histological lesions of lungs were scored according to the thicken degrees of alveolar septum that comparing to the mock infection mice and ranged from 0 (normal) to 3 (severe).

### Statistical Analysis

The results are representative of three independent experiments. The data are presented as mean ± SEM or mean ± SD as mentioned in each figure legend. Comparisons between the two groups were performed by unpaired Student’s *t* test, whereas multiple group data were analyzed by ANOVA, followed by Bonferroni *post hoc* test. Statistically significant and very significant results were defined as *p* < 0.05 and *p* < 0.01.

## Results

### Porcine Circovirus Type 2 Upregulated Interleukin-10 Expression to Promote Persistent Infection in Mice

**Figure 1:**
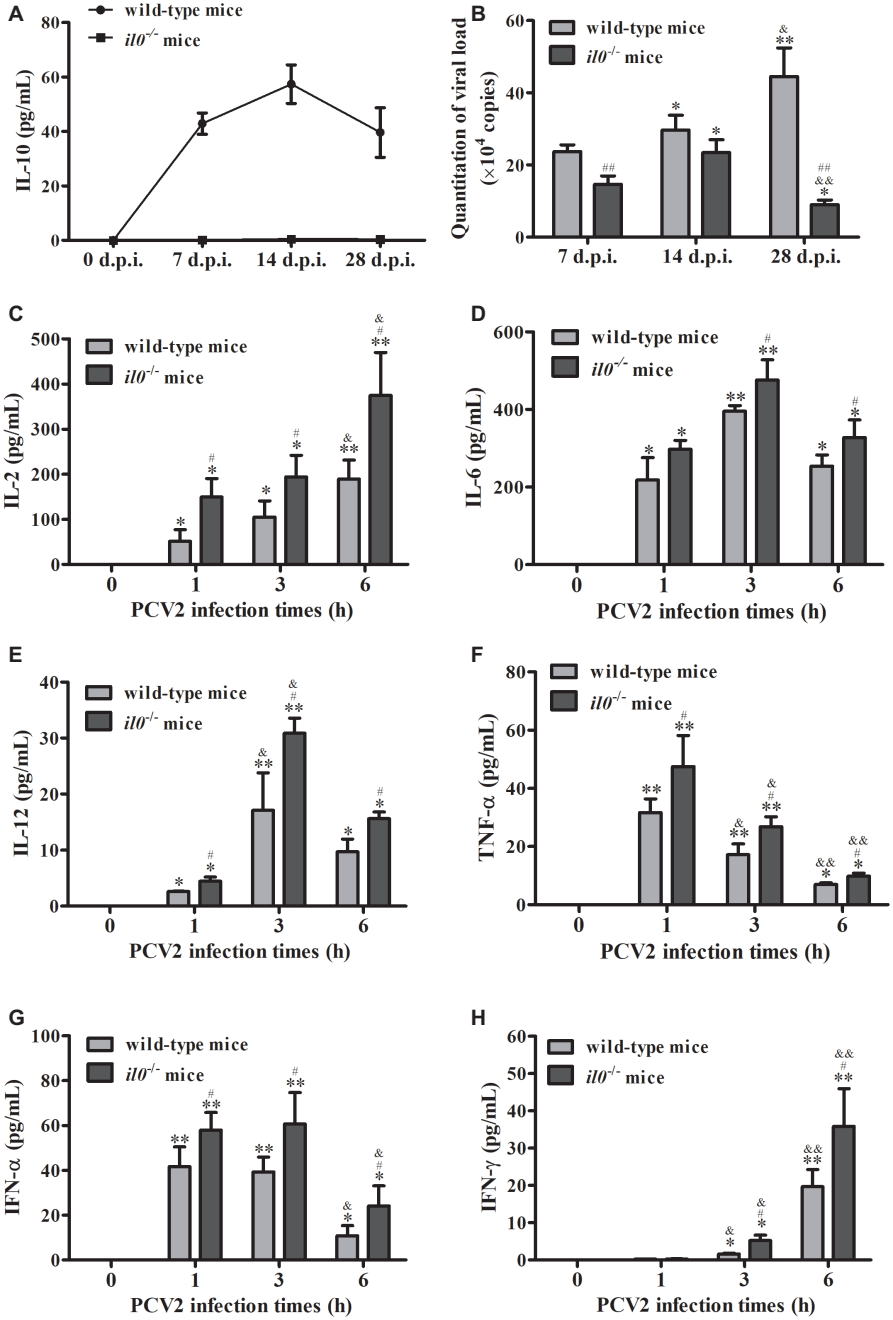
PCV2 infection induces IL-10 production to promote viral replication. Wild-type mice and *il10*^−/−^ mice were infected with PCV2, and samples were collected at 7, 14, and 28 d.p.i. **(A)** The serum IL-10 expression was measured by ELISA *n* = 15. **(B)** The PCV2 copy numbers in lungs were detected by qPCR. The data are presented as mean ± SEM of three independent experiments *n* = 15. **(C–H)** Other groups of wild-type mice and *il10*^−/−^ mice were infected with PCV2. The IL-2, IL-6, IL-12, TNF-α, IFN-α, and IFN-γ production were measured by ELISA at 0, 1, 3, and 6 h.p.i., respectively. The value of the cytokines in wild-type mice is the value of PCV2-infected wild-type mice minus the value of mock-infected wild-type mice; the value of pro-inflammatory cytokines in *il10*^−/−^ mice is the value of PCV2-infected *il10*^−/−^ mice minus the value of mock-infected *il10*^−/−^ mice. The production of cytokines at 0 h post-infection was undetectable. The data are presented as mean ± SEM of three independent experiments *n* = 15 mice. **(B)** ^*^p < 0.05, ^**^p < 0.01 versus same group at 7 d.p.i.; ^&^*p* < 0.05, ^&&^*p* < 0.01 versus same group at 14 d.p.i.; ^##^*p* < 0.01 versus wild-type mice at same infection time. **(C–H)** ^*^p < 0.05, ^**^p < 0.01 versus same group at 0 h post-infection; ^&^*p* < 0.05, ^&&^*p* < 0.01 versus same group at 1 h post-infection; ^#^*p* < 0.05 versus wild-type mice at same infection time.

To explore the roles of IL-10 during PCV2 infection, we infected wild-type C57BL/6 mice and *il10*^−/−^ mice with mock or PCV2. Detection of the IL-10 level in peripheral blood showed that PCV2 infection significantly upregulated the IL-10 production from 7 d.p.i., and the IL-10 production were raised to the highest level at 14 d.p.i. and then reduced (Figure 1A). No IL-10 production was detected in *il10*^−/−^ mice (Figure 1A). In the lungs of wild-type mice, PCV2 level was progressively accumulated from 7 to 28 d.p.i.; in the lungs of *il10*^−/−^ mice, PCV2 level increased from 7 to 14 d.p.i., then dramatically decreased at 28 d.p.i. (Figure 1B). However, PCV2 level was significantly higher in the lung of PCV2-infected wild-type mice than in the lung of PCV2-infected *il10*^−/−^ mice at 7 and 28 d.p.i., particularly at 28 d.p.i. (Figure 1B). In addition, the levels of pro-inflammatory cytokines (IL-2, IL-6, IL-12, TNF-α, IFN-α, IFN-γ) were higher in *il10*^−/−^ mice than in wild-type mice at 1, 3, and 6 h post infection (Figures 1C–H). These results suggest that IL-10 plays an important role in controlling the viral replication level at the late phase of PCV2 infection.

### Interleukin-10 Deficiency Increases Porcine Circovirus Type 2-Specific Antibodies in Infected Mice

**Figure 2:**
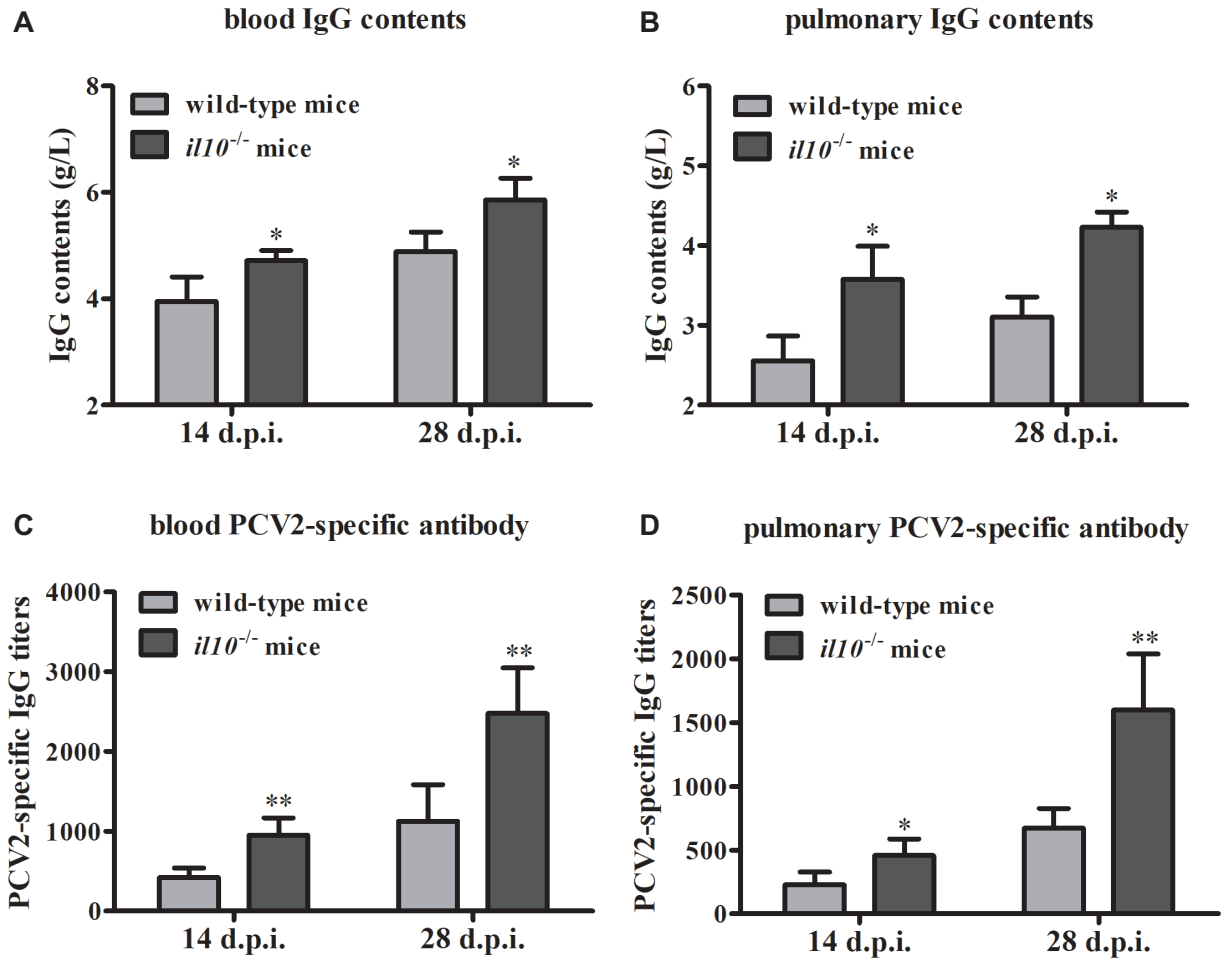
IL-10 deficiency increases PCV2-specific antibody production in PCV2-infected mice. **(A,B)** The IgG contents of blood and lung were measured by automatic biochemical analyzer, the IgG value is the value of PCV2-infected wild-type mice minus the value of mock-infected wild-type mice, or the value of PCV2-infected *il10*^−/−^ mice minus the value of mock-infected *il10*^−/−^ mice. The data are presented as mean ± SEM of three independent experiments. *n* = 15 mice. **(C,D)** The PCV2-specific antibodies in blood and lung were detected by ELISA. The data are presented as mean ± SEM of three independent experiments. *n* = 15 mice. ^*^p < 0.05, ^**^p < 0.01 versus PCV2-infected wild-type mice at same infection time.

To figure out the role of IL-10 during the anti-PCV2 response in mice, we collected the serums from wild-type mice and *il10*^−/−^ mice, respectively. The IgG content measurement results showed that PCV2 infection induced more IgG in *il10*^−/−^ mice than in wild-type mice in either blood or lung (Figures 2A,B). At the same time, PCV2 infection induced more PCV2-specific antibodies in blood and lungs of *il10*^−/−^ mice than in wild-type mice (Figures 2C,D). These results suggest that IL-10 deficiency might promote the increase of PCV2-specific antibodies in PCV2-infected mice.

### Interleukin-10 Deficiency Enhances the Infiltration of CD45^+^ Cells, CD4^+^ and CD8^+^ T Cells in the Lung of Porcine Circovirus Type 2-Infected Mice

**Figure 3:**
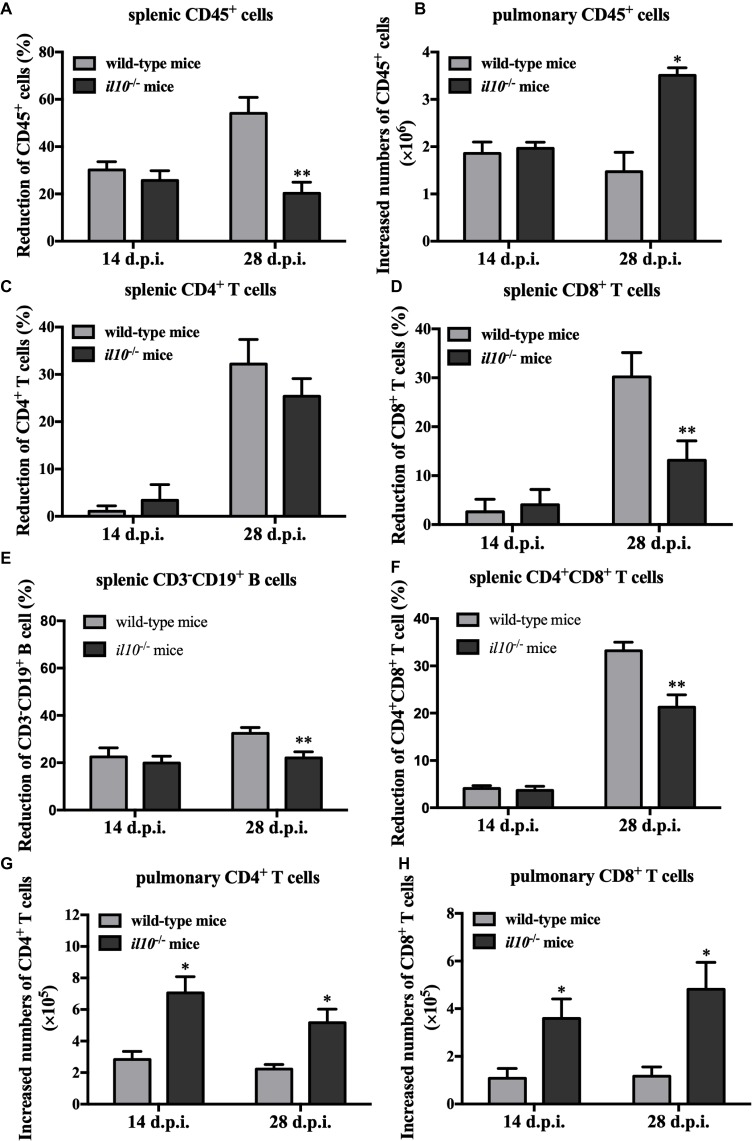
IL-10 deficiency decreases splenic CD45^+^ cells’ depletion and promoted pulmonary CD4^+^ and CD8^+^ T cells’ infiltration in PCV2-infected mice. Wild-type mice and *il10*^−/−^ mice were infected with PCV2, and CD45^+^ cells, CD3^+^CD4^+^ and CD3^+^CD8^+^ lymphocytes from spleen and lung were measured by flow cytometry. **(A)** The reduction percentages of splenic CD45^+^ cells [(splenic CD45^+^ cells’ number in mock-infected mice − splenic CD45^+^ cells’ number in PCV2-infected mice)/splenic CD45^+^ cells’ number in mock-infected mice × 100%] were calculated. The results are expressed as splenic CD45^+^ cell decreased rate mean ± SEM of three independent experiments *n* = 15 mice. **(B)** The increased numbers of pulmonary CD45^+^ cells (pulmonary CD45^+^ cells’ number in PCV2-infected mice − pulmonary CD45^+^ cells’ number in mock-infected mice) were calculated. The results are expressed as pulmonary CD45^+^ cell increased numbers mean ± SEM of three independent experiments *n* = 15 mice. **(C)** The percentages of CD4^+^ T cells’ reduction in spleen. **(D)** The percentages of CD8^+^ T cells’ reduction in spleen. **(E)** The percentages of CD3^−^CD19^+^ B cells in spleen. **(F)** The percentages of CD4^+^CD8^+^ T cells in spleen. **(G)** The increased numbers of CD4^+^ T cells in lungs. **(H)** The increased numbers of CD8^+^ T cells in lungs. The data are presented as mean ± SD. *n* = 5 mice. ^*^p < 0.05, ^**^p < 0.01 versus PCV2-infected wild-type mice at same infection time.

To make clear the role of IL-10 in PCV2-induced depletion of lymphocytes, we measured the numbers of total lymphocytes (CD45^+^ cells) in spleens and lungs of the PCV2-infected wild-type and *il10*^−/−^ mice. As the results showed, in wild-type mice, PCV2 infection significantly reduced the counts of splenic CD45^+^ cells at both 14 and 28 d.p.i., compared to the mock infection. The reduction percentages of splenic CD45^+^ cells in PCV2-infected wild-type mice were 30.19 and 54.08% at 14 and 28 d.p.i., respectively. In *il10*^−/−^ mice, PCV2 infection also reduced the counts of splenic CD45^+^ cells at 14 d.p.i. (25.68%) and 28 d.p.i. (20.28%) relative to the mock infection (Figure 3A). Notably, at 28 d.p.i., the reduction rate of splenic CD45^+^ cells in *il10*^−/−^ mice was much less than in wild-type mice (Figure 3A), suggesting that the presence of IL-10 might promote the depletion of splenic CD45^+^ cells at the late phase of PCV2 infection. In lungs, the numbers of CD45^+^ cells were significantly increased by PCV2 infection in both wild-type and *il10*^−/−^ mice, and the increased number of CD45^+^ cells did not show significant difference between wild-type and *il10*^−/−^ mice at 14 d.p.i. (Figure 3B). However, at 28 d.p.i., the increased number of CD45^+^ cells in the lung of *il10*^−/−^ mice was higher than in the lung of wild-type mice (Figure 3B), suggesting that the presence of IL-10 prevents the transfer of CD45^+^ cells into the lung at the late phase of PCV2 infection.

Given IL-10 was associated with the depletion of splenic lymphocytes and the infiltration of pulmonary lymphocytes, we further analyzed the relationship of CD4^+^ and CD8^+^ T cells changes with IL-10 in spleen and lung. At 14 days post-PCV2 infection, PCV2 infection-induced splenic CD4^+^ and CD8^+^ T cell reduction rates were lower than 5% in both wild-type and *il10*^−/−^ mice, and there was no difference between these two kinds of mice (Figures 3C,D). However, at 28 days post-PCV2 infection, PCV2 infection-induced splenic CD4^+^ T cell reduction rates were about 30% in both wild-type and *il10*^−/−^ mice (Figure 3C), and it induced more splenic CD8^+^ T cell reduction in wild-type than in *il10*^−/−^ mice (30.18 vs. 13.16%) (Figure 3D). These results suggest that IL-10 deficiency might prevent the reduction of CD8^+^ T cells induced by PCV2 at the later phase of infection.

We also checked the changes of CD3^−^CD19^+^ B cells and CD4/CD8 double positive cells in spleens of PCV2-infected wild-type mice and *il10*^−/−^ mice. Results showed that PCV2 infection led to the reduction of CD3^−^CD19^+^ B cells in spleens of wild-type mice and *il10*^−/−^ mice at 14 and 28 d.p.i. compared to mock infection. The PCV2 infection-induced splenic CD3^−^CD19^+^ B cell reduction was significantly lower in *il10*^−/−^ mice than in wild-type mice at 28 d.p.i. (Figure 3E). Similar to the changes of CD4^+^ and CD8^+^ T cells, PCV2 infection did not induce a significant reduction of splenic CD4 and CD8 double positive T cells (CD4^+^CD8^+^ T cells) at 14 d.p.i. (Figure 3F). However, at 28 d.p.i., PCV2 infection induced a significant splenic CD4^+^CD8^+^ T cell reduction, which was significantly less in *il10*^−/−^ mice than in wild-type mice (Figure 3F).

In lungs, PCV2 infection increased the numbers of pulmonary CD4^+^ and CD8^+^ T cells in either wild-type mice or *il10*^−/−^ mice (Figures 3G,H). Meanwhile, PCV2 infection induced more pulmonary CD4^+^ and CD8^+^ T cells in *il10*^−/−^ mice than in wild-type mice (Figures 3E,F). These results demonstrate that IL-10 deficiency enhances the infiltration of CD4^+^ and CD8^+^ T cells in lung during PCV2 infection.

### Interleukin-10 Deficiency Enhances CCL3, CXCL9, and CXCL10 Expression in Lungs of Porcine Circovirus Type 2-Infected Mice

**Figure 4:**
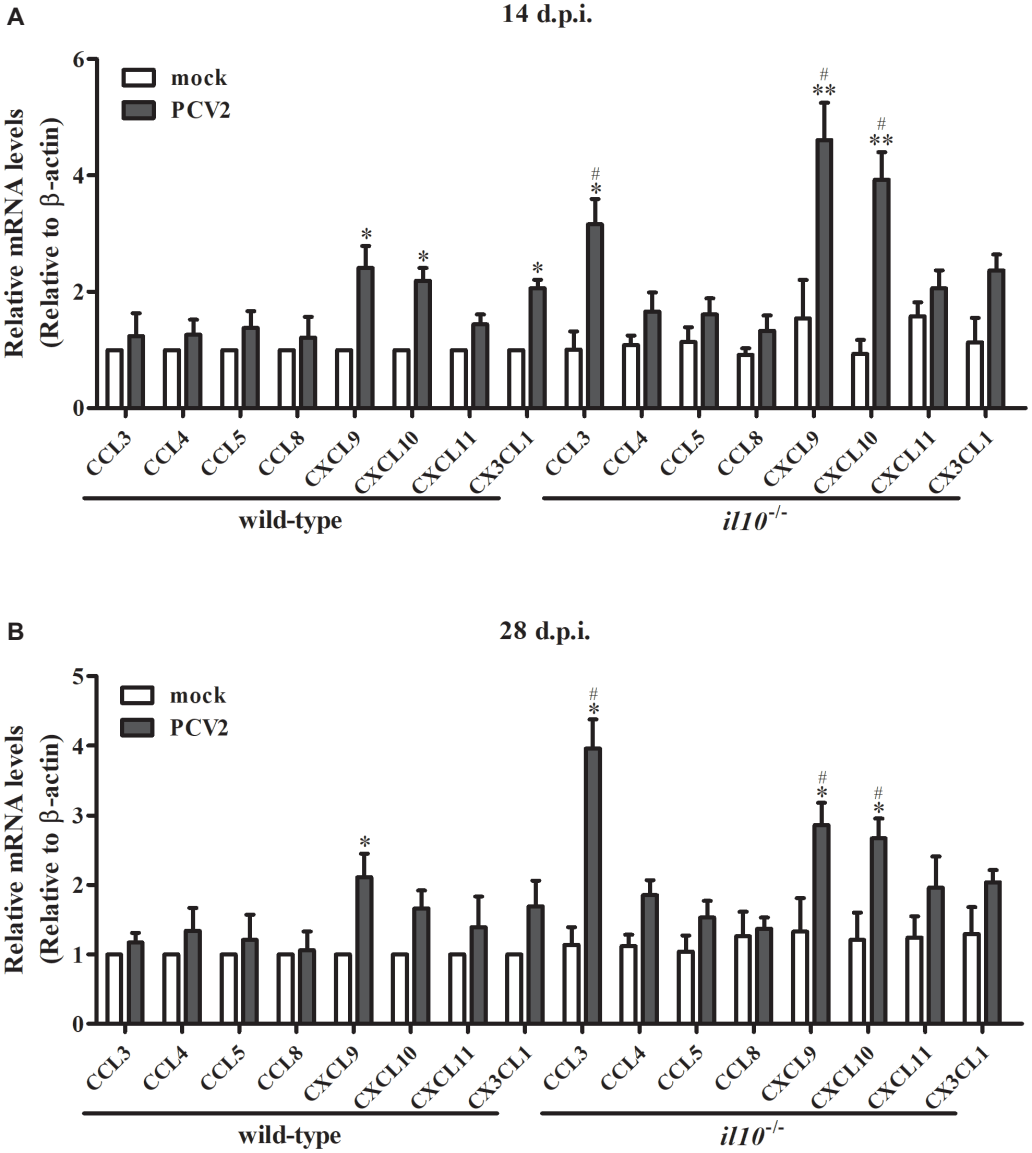
IL-10 deficiency enhances CCL3, CXCL9, and CXCL10 expression in lungs of PCV2-infected mice. Wild-type mice and *il10*^−/−^ mice were infected with PCV2, and the expression of chemokines indicated in the left lung of the PCV2-infected mice was measured by qPCR at 14 d.p.i. **(A)** and 28 d.p.i. **(B)**, respectively. The levels of chemokine expressed in lungs of mock-infected wild-type mice were settled as 1. The data are presented as mean ± SEM of three independent experiments. *n* = 15 mice. ^*^p < 0.05, ^**^p < 0.01 versus mock infection; ^#^*p* < 0.05 versus the same chemokines in PCV2-infected wild-type mice.

To further figure out the transfer enhancement mechanism of T cells in the lung of *il10*^−/−^ mice, several chemokines contributing to T cells’ migration into lung were measured. At 14 d.p.i., PCV2 infection upregulated CXCL9 and CXCL10 in both wild-type mice and *il10*^−/−^ mice, and the expression of PCV2-induced CXCL9 and CXCL10 was significantly higher in *il10*^−/−^ mice than in wild-type mice (Figure 4A). While PCV2 infection could also increase CX3CL1 expression in wild-type mice and CCL3 expression in *il10*^−/−^ mice, respectively (Figure 4A). At 28 d.p.i., PCV2 infection only increased CXCL9 in wild-type mice, but still markedly increased CCL3, CXCL9, and CXCL10 expression in *il10*^−/−^ mice, and revealed a higher level than that in wild-type mice (Figure 4B). These results indicate that PCV2 infection mainly induces CXCL9 and CXCL10 to promote the migration of T cells to lung, while IL-10 deficiency can enhance the expression of CCL3, CXCL9, and CXCL10 that further promote T cells’ migration to lung.

### Interleukin-10 Deficiency Alleviates Viral Replication and Lung Lesion in Porcine Circovirus Type 2-Infected Mice *via* Enhancing Pulmonary T Cell Infiltration

**Figure 5:**
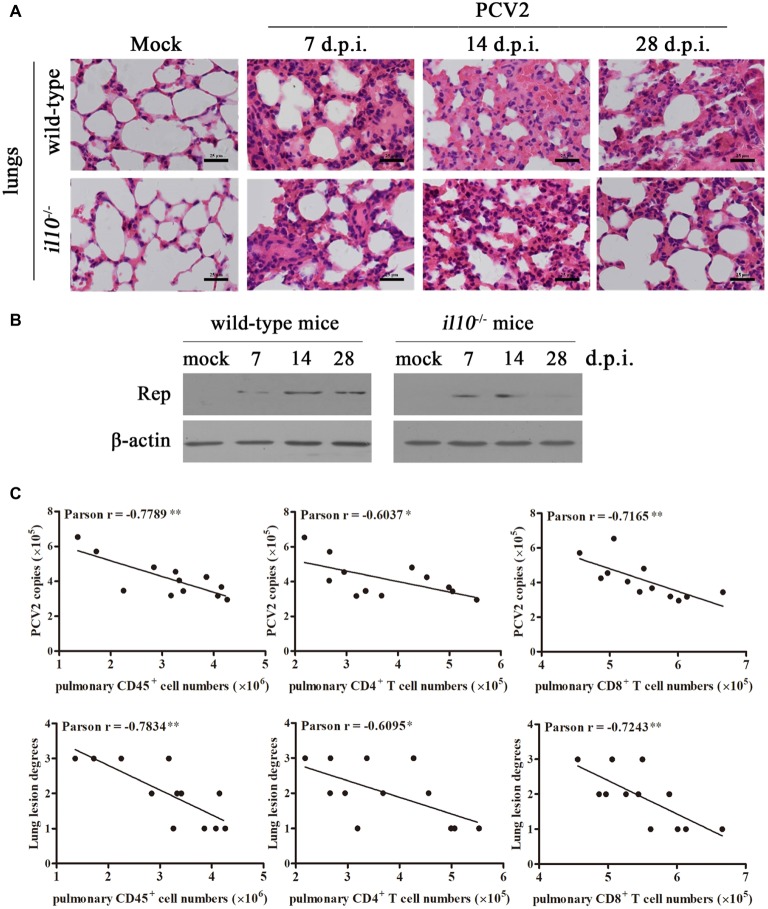
IL-10 deficiency alleviates virus replication and PCV2-induced pathological changes through increasing T cell infiltration. **(A)** Wild-type mice and *il10*^−/−^ mice were infected with PCV2, the lungs were collected, and then paraffin sections were stained with H&E for observation at 7, 14, and 28 d.p.i. Bar = 25 μm. **(B)** Wild-type mice and *il10*^−/−^ mice were infected with PCV2, the PCV2 Rep expression in the lungs was measured by western blot at 7, 14, and 28 d.p.i. **(C)** Mixed feeding wild-type mice and *il10*^−/−^ mice were infected with different doses of PCV2. At 28 d.p.i., the numbers of lymphocytes in lung of PCV2-infected mice were counted, and the correlation of the pulmonary lymphocyte numbers and the viral number (upper panel) or lung lesion scores (lower panel) analyzed. ^*^p < 0.05, ^**^p < 0.01 demonstrated that cell number is inversely correlated with PCV2 copies or lung lesion degree.

To determine the relationship between IL-10-regulated T cell subsets’ changes and pathological changes in PCV2-infected mice, we further observed the pathological changes of lung in both wild-type mice and *il10*^−/−^ mice after PCV2 infection. At 7, 14, and 28 days post-PCV2 infection, both wild-type mice and *il10*^−/−^ mice exhibited a thickened alveolar septum and increased pulmonary infiltration of inflammatory cells (Figure 5A, Table 2), suggesting PCV2 infection could also induce interstitial pneumonia in mice. However, notably, in wild-type mice, PCV2-induced lesions in lung were continuing to worsen at 7, 14, and 28 d.p.i., whereas PCV2-induced lesions did not exhibit a worse tendency in the lung of *il10*^−/−^ mice (Figure 5A, Table 2). At 7 d.p.i., the pathological changes of lung did not show apparent difference between wild-type mice and *il10*^−/−^ mice; at 14 and 28 d.p.i., wild-type mice exhibited a more severe interstitial pneumonia than *il10*^−/−^ mice (Figure 5A, Table 2). To further figure out the relationship of pathological changes with PCV2 replication, the expression of PCV2 Rep in the lungs was measured by western blot. Results showed, in wild-type mice, the PCV2 Rep expression was continuously increased from 7 to 28 d.p.i., while the Rep expression levels increased from 7 to 14 d.p.i., then decreased at 28 d.p.i. in the lung of PCV2-infected *il10*^−/−^ mice (Figure 5B). Meanwhile, the Rep expression level was significantly higher in wild-type mice than in *il10*^−/−^ mice at 28 d.p.i. (Figure 5B). These results suggest that IL-10 deficiency could decrease the replication of PCV2 and alleviate PCV2-induced lung lesion.

**Table 2:** Comparison of histological lesion score of lungs from PCV2-infected wild-type mice and *il10*^−/−^ mice.

Group	Mice no.^a^	Wild-type mice lung^b^	*il10*^−/−^ mice lung^b^
7 d.p.i.	I	2	1
	II	2	2
	III	2	2
14 d.p.i.	I	3	2
	II	3	2
	III	2	2
28 d.p.i.	I	3	1
	II	3	2
	III	3	3

a *One part of the left lungs from three out of five PCV2-infected mice was used for histopathological examination*.

b *The score was according to the thicken degrees of alveolar septum that comparing to mock infection mice and ranged from 0 (normal) to 3 (severe)*.

To further confirm the relationships between pulmonary lymphocyte numbers and lung lesion degrees as well as PCV2 replications, mixed feeding mice (wild-type and *il10*^−/−^ mice) were infected with different doses of PCV2. At 28 d.p.i., the lung lesion degrees were scored according to the thickened alveolar septum. The numbers of pulmonary CD45^+^ cells, CD4^+^ T cells, and CD8^+^ T cells were measured by flow cytometry, and the PCV2 copies were detected by qPCR. The relationships of these data were analyzed by Pearson correlation analysis. Results showed that the numbers of pulmonary CD45^+^ cells, CD4^+^ T cells, and CD8^+^ T cells were all inversely associated with the lung lesion degree, as well as the replication levels of PCV2 (Figure 5C). Compared to pulmonary CD4^+^ T cells, pulmonary CD45^+^ cells and CD8^+^ T cells were more highly correlated with the lung lesion degree and PCV2 replication levels (Figure 5C). Taken together, these results demonstrate that IL-10 suppresses the infiltration of pulmonary T lymphocyte to promote viral replication and aggravate lung lesion during PCV2 infection.

## Discussion

Considering of the clear genetic background, convenience, and low cost, mice are excellent models for research, especially the genetic knockout mice. Besides swine, PCV2 is reported to infect mice (Kiupel et al., 2001; Csagola et al., 2008; Deng et al., 2013; de Castro et al., 2015; Wang et al., 2017). In this study, we infected mice with PCV2 and results showed that PCV2 infection could induce the similar pathological changes in mice as that in pigs, which performed as IL-10 production, splenic lymphocytes depletion, and pulmonary lymphocytes infiltration (Segalés, 2012). These results were confirmed by other studies that PCV2 infection leads to lymphoid depletion and suppression of CD4^+^ T cell and CD8^+^ T cell functions in BALB/c mice, and interstitial pneumonia in C57BL/6 mice (Kiupel et al., 2001; Fu et al., 2014; Wang et al., 2017). Using *il10*^−/−^ mice, we found that IL-10 deficiency partly prevents the depletion of splenic lymphocytes and promotes the infiltration of pulmonary lymphocytes. These results demonstrate that mice could be a suitable and cheaper model to study the pathogenesis of PCV2.

IL-10 is proved to be upregulated in pigs by PCV2 infection, and the upregulated IL-10 was reported to associate with the thymic depletion, repress IL-12 production, and mainly localize in T cell rich areas in PCV2-infected lymphoid tissues (Darwich et al., 2003; Kekarainen et al., 2008; Doster et al., 2010; Du et al., 2016). In this study, we confirmed that PCV2 could also induce IL-10 production in mice to promote infection, as well as in piglets (Du et al., 2016). Deficiency of IL-10 improved the anti-virus ability of the mice at the early phase of infection through increasing either pro-inflammatory cytokines’ (IL-2, IL-6, IL-12, TNF-α, IFN-α, IFN-γ) production or PCV2-specific antibodies. Meanwhile, PCV2 could employ IL-10 to promote splenic lymphocytes depletion and to suppress the pulmonary T cell infiltration, leading to a lower virus clearance ability at the later phase of PCV2 infection, which caused a significantly increased PCV2 viral load in infected tissue and further induced more severe tissue lesion. Since IL-10 inhibits the proliferation of lymphocytes (O’Farrell et al., 1998; Pino-Martinez et al., 2019), we found that IL-10 deficiency led to largely prevention of the PCV2-induced splenic lymphocytes (CD45^+^ cells) depletion. Considering this, we think IL-10 might play an important but not the only role in PCV2 infection-induced lymphocytes depletion, which is the characteristic lesion of PCVD. Besides, IL-10 deficiency also improved PCV2-induced CD45^+^ cell infiltration in lung, the place that the virus initially infected. The PCV2 infection-induced IL-10 would help the virus escaping from host clearance to cause persistent infection.

T cells play a central role in host immune responses. Activation of T cells leads to action of T-helper cells (CD4^+^) that contribute to increase of antibody responses and enhancement of antibody production by B cells. Activation of T cells can also lead to the development of cell-mediated immune response through cytotoxic T cells (CD8^+^) (Pross, 2007). In this study, we found that PCV2 induced the reduction of splenic CD4^+^ and CD8^+^ T cells at late phase of infection in wild-type mice, and increased the pulmonary CD4^+^ and CD8^+^ T cells. In *il10*^−/−^ mice, the PCV2-induced splenic CD8^+^ T cell reduction was distinctly prevented, and IL-10 deficiency notably enhanced the infiltration of CD4^+^ and CD8^+^ T cells in lungs. Besides, CD4 and CD8 double positive T cells (CD4^+^CD8^+^ T cells) are important cell phenotypes during viral infection (Nascimbeni et al., 2004). We found that IL-10 deficiency significantly inhibited the PCV2-induced reduction of CD4^+^CD8^+^ T cells in spleens of mice. Combined with the results we previously found that PCV2 infection suppressed the host Th1 cells’ responses to other pathogens (Du et al., 2018), we infer that PCV2 infection leads to dysfunction of T cells through IL-10 production in mice.

Chemokines constitute a special family of small cytokines that are able to guide migration and residence of immune cells (Arango Duque and Descoteaux, 2014). Since the different counts of T cells in lungs and spleens during PCV2 infection, we considered chemokines might participated in the process. Chemokines, based on the conserved cysteine motifs, are usually classified into sub-families: C, CC, CXC, and CX3C (Susek et al., 2018). CXCL9, CXCL10, and CXCL11, which were commonly produced by local cells in inflammatory lesions, are able to attract Th1 cell (Tokunaga et al., 2018). CXCL9, also known as monokine induced by gamma interferon (MIG), is a strong T cell chemoattractant to the site of inflammation (Ding et al., 2016). CXCL10, interferon gamma-induced protein 10, also serves to attract T cells (Rogers et al., 2018). In this study, we found that PCV2 infection upregulated the expression of CXCL9 and CXCL10 to migrate T cells to lungs in mice. IL-10 deficiency resulted in further enhancement of CXCL9, CXCL10, as well as CCL3 expression at 14 and 28 d.p.i. The enhanced expression of chemokines might result in more CD4^+^ T cells and CD8^+^ T cell infiltration in lungs of *il10*^−/−^ mice, which would benefit virus clearance. However, how PCV2 infection controls the expression of chemokines in lungs needs to be further explored.

Overall, our findings demonstrate the important roles of IL-10 played in PCV2 infection, lymphocytes alteration, and lung lesions. These findings may provide further insight into the mechanism of immune responses and pathogenesis during PCV2 infection.

## Data Availability

The raw data supporting the conclusions of this manuscript will be made available by the authors, without undue reservation, to any qualified researcher.

## Author Contributions

YH and QD contributed to the conception and design of the study. QD and HZ performed the experiments. MH and XZ operated the software. JH, BC, and XY validated the results. QD wrote the first draft of the manuscript. YH and DT contributed to manuscript revision. All authors read and approved the submitted version.

### Conflict of Interest Statement

The authors declare that the research was conducted in the absence of any commercial or financial relationships that could be construed as a potential conflict of interest.

## Supplementary Material

SUPPLEMENTARY FIGURE S1 The gate strategies of this study.
